# *Trypanosoma congolense* Infections: Induced Nitric Oxide Inhibits Parasite Growth In Vivo

**DOI:** 10.1155/2011/316067

**Published:** 2011-04-05

**Authors:** Wenfa Lu, Guojian Wei, Wanling Pan, Henry Tabel

**Affiliations:** ^1^College of Animal Science and Technology, Jilin Agricultural University, Changchun 130118, China; ^2^Department of Veterinary Microbiology, Western College of Veterinary Medicine, University of Saskatchewan, 52 Campus Drive, Saskatoon, SK, Canada S7N 5B4

## Abstract

Wild-type (WT) C57BL/6 mice infected intraperitoneally with 5 × 10^6^ *Trypanosoma congolense* survive for more than 30 days. C57BL/6 mice deficient in inducible nitric oxide synthase (iNOS^−/−^) and infected with 10^3^ or 5 × 10^6^ parasites do not control the parasitemia and survive for only 14 ± 7 or 6.8 ± 0.1 days, respectively. Bloodstream trypanosomes of iNOS^−/−^ mice infected with 5 × 10^6^ *T. congolense* had a significantly higher ratio of organisms in the S+G2+M phases of the cell cycle than trypanosomes in WT mice. We have reported that IgM anti-VSG-mediated phagocytosis of *T. congolense* by macrophages inhibits nitric oxide (NO) synthesis via CR3 (CD11b/CD18). Here, we show that during the first parasitemia, but not at later stages of infection, *T. congolense*-infected CD11b^−/−^ mice produce more NO and have a significantly lower parasitemia than infected WT mice. We conclude that induced NO contributes to the control of parasitemia by inhibiting the growth of the trypanosomes.

## 1. Introduction

*Trypanosoma congolense* is a protozoan pathogen of cattle and other livestock. The parasite causes N'gana in livestock, one form of the disease complex collectively known as African trypanosomiases [1]. In the mammalian host, the whole parasite is covered with a glycoprotein coat of a single molecular species, called variant surface glycoprotein (VSG) [2]. In host defense against infection, macrophages play an important role through their ability to remove specific substances from the blood stream via various receptors, such as complement receptors, Fc-receptors, scavenger receptors, and mannose receptors [3, 4]. The control of parasitemia in African trypanosomiasis is mediated by at least two known mechanisms: (1) antibody-mediated phagocytosis [5–9] and (2) to a lesser degree, by antibody/complement-mediated lysis [10–13]. A third mechanism, that is, release of trypanotoxic NO by macrophages has been demonstrated in vitro for *T. brucei* and *T. congolense* [14–18]. The role of NO in vivo has been controversial. We had speculated that NO might be involved in the control of *T. congolense* infections [9]. There is evidence that NO does not contribute to control of *T. brucei* in vivo [19, 20] but does contribute to control of *T. congolense* infections [21, 22]. We found that IgG2a anti-VSG antibody-mediated phagocytosis of *T. congolense* enhances the synthesis of NO by macrophages, whereas IgM anti-VSG antibody-mediated phagocytosis inhibited synthesis of NO [7, 14]. The inhibition of NO synthesis apparently increased with increasing amounts of IgM anti-VSG [14]. We further observed that macrophages of CD11b^−/−^ mice were much less efficient in phagocytosis of *T. congolense* via opsonization by IgM anti-VSG and complement than macrophages of WT mice, indicating that IgM anti-VSG-mediated phagocytosis is greatly enhanced by complement receptor CR3 [7]. Macrophages of CD11b^−/−^ mice, however, produced much more NO in response to IgM anti-VSG- mediated phagocytosis than macrophages of WT mice. It appeared that, during IgM antibody-mediated phagocytosis, the induced NO synthesis by WT macrophages was inhibited by the binding of a parasite component to CD11b [7]. In other words, it appears that the parasite has developed a mechanism trying to evade the deleterious effect of NO. We predicted that CD11b-deficient (CD11b^−/−^) mice might control *T. congolense* infection via NO more efficiently than infected WT mice [7]. Here, we confirm the finding of the involvement of induced NO synthesis in resistance to *T. congolense* infections [21, 22] and provide data that induced NO inhibits the multiplication of *T. congolense *in vivo.

## 2. Materials and Methods

### 2.1. Mice

Eight-to 10-week-old, female mice deficient in inducible nitric oxide synthase (iNOS^−/−^) were purchased from Jackson laboratories (genetic background: C57BL/6; strain name: B6.129P2-NOS2). Eight-to 10-week-old, female CD11b^−/−^ mice were obtained from Jackson Laboratories (genetic background: C57BL/6; strain name: B6.129S4-Itgam^tm1Myd^/J; stock number: 003991). Eight-to 10-week-old, female C57BL/6 mice and 5- to 8-week-old, female, Swiss white mice (CD1) were purchased from the Animal Resource Center of the University of Saskatchewan (Saskatoon, Canada). The mice were kept in polycarbonate cages on sawdust and allowed free access to food and water throughout the experiments, according to the recommendations of the Canadian Council of Animal Care.

### 2.2. Parasites, Infections, Parasitemia, and Survival Time

*T. congolense*, Trans Mara strain, variant antigenic type (VAT) TC13 was used in this study. The origin of this parasite strain has been previously described [23]. Frozen stabilates of parasites were used for infecting CD1 mice immunosuppressed with cyclophosphamide, and passages were made every third day as described previously [23]. The parasites purified from the blood of infected CD1 mice by DEAE-cellulose chromatography were used for infection [24]. For experiments, mice were infected intraperitoneally (i.p.) with 10^3^ or 5 × 10^6^ parasites. To determine the degree of parasitemia, 10 *μ*l of blood were collected from the mouse tail. Parasites were counted in diluted blood samples by the use of a hemocytometer. Mice showing signs of the terminal stage were euthanized.

### 2.3. Spleen Cell Cultures and Cytokine Assays

Spleen cells of individual mice were cultured in 24-well plates at an optimal cell density (5 × 10^6^/mL). Supernatant fluids of the cell cultures were harvested after incubation for 48 h. Culture supernatant fluids were centrifuged for 10 min at 1000 × g to remove cellular debris, transferred to new tubes, and stored at –80°C until analysis. The levels of cytokines (TNF-*α*, IL-12p40, MCP-1, IFN-*γ*, and IL-*10*) in the culture supernatant fluids were determined by routine sandwich ELISA by using Immulon-4 plates and Opt EIA ELISA kits (BD Biosciences Pharmingen, San Diego, CA), according to the manufacturer's protocol. Each sample of the cell culture fluids was tested for cytokines in triplicate.

### 2.4. Measurement of Nitrite Production

Nitrite concentrations in the culture fluids harvested after 48 hr were determined by the “Griess reagent system” (Promega, Madison, WI, U.S.A.) as described previously [14]. Briefly, 50 *μ*L of culture supernatant fluids were incubated with an equal volume of sulfanilamide solution (1% sulfanilamide (Sigma) in 5% phosphoric acid (Sigma)) for 5–10 min at room temperature, protected from light. Then 50 *μ*l of the NED (Sigma) (0.1% N-1-napthylethylenediamine dihydrochloride in water) was added and incubated for another 10 min at room temperature, protected from light. The absorbance was measured at 550 nm within 30 min in a micro-ELISA reader. Nitrite levels were determined by comparison with a sodium nitrite (Sigma) standard curve. The sensitivity of the detection limit of this assay was 2.5 *μ*M.

### 2.5. Inhibition of Inducible Nitric Oxide Synthase

Some groups of mice were injected intravenously (i.v.) with L-N6-(1-imminoethyl) lysine (L-NIL) (Sigma, Oakville, ON), a specific inhibitor of iNOS [25], that is, with 20 or 40 mg/kg body weight-1, 1, 2, and 3 days after infection.

### 2.6. Use of Flow Cytometry for DNA Analysis of Trypanosomes

Ten microliters of blood were collected from the tail of infected mice (2–6 days post infection) and diluted to 1 : 50 in an ice-cold solution of tris-buffered saline glucose and 10 U/mL heparin (TBSG/heparin) [24]. These parasite-containing samples were washed 1 × with ice-cold TBSG, that is, centrifuged at 1000 × g at 4°C for 10 min. About 450 *μ*L of the supernatants were removed. The sedimented cells were resuspended in the remaining fluid with the use of a pipette. The cells were fixed in ethanol by adding 500 *μ*L 40% ice-cold ethanol/PBS, mixed vigorously with a vortex shaker for 30 s, and kept on ice for at least 1 h. Then the samples were spun at 1000 × g for 10 min and about 400 *μ*L of the supernatant fluids were removed, leaving about 100 *μ*L of ethanol/cells in the tube. The cells were resuspended in the residual ethanol. Then, 1000 *μ*l of propidium iodide (P.I.) staining solution (Molecular Probes, Eugene, Oregon, USA) was added to each tube and carefully mixed by a vortex shaker. The samples were incubated at room temperature in the dark. Analysis of the samples for DNA content by FACS was carried out within 24 hours [26, 27]. Trypanosomes purified from immunosuppressed, infected CD1 mice were analyzed by FACS to establish the appropriate gating.

### 2.7. Statistical Analysis

Data are presented as means ± standard error (SE). Analysis of Variance (ANOVA) was carried out using Excel software (Microsoft, Santa Monica, CA, USA). A *P* value < .05 was considered statistically significant.

## 3. Results

### 3.1. Induced Nitric Oxide Reduces Parasitemia and Enhances Survival of *T. congolense*-Infected Mice

**Figure 1:**
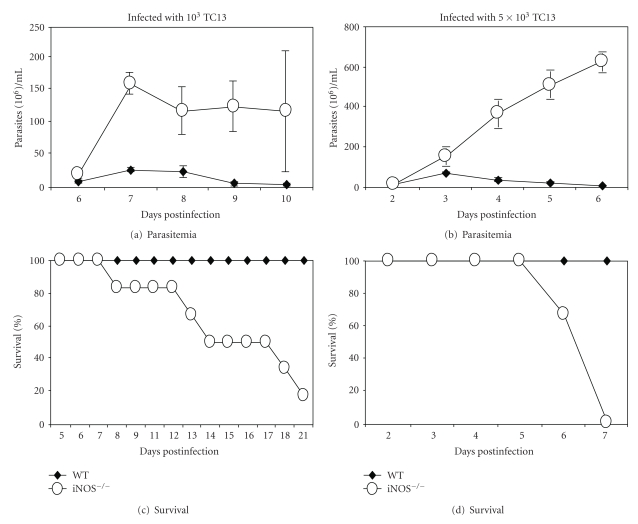
iNOS^−/−^ mice are highly susceptible to infections by *T. congolense*: enhanced parasitemia and reduced survival time. Groups of 5 to 10 WT or iNOS^−/−^ C56BL/6 mice were infected with low (10^3^) or high (5 × 10^6^) doses of *T. congolense* TC13 and parasitemia and survival time were determined. The initial parasitemia is about 5-fold higher in iNOS^−/−^ mice infected with 10^3^ s*T. congolense* than in infected wild-type (WT) mice (a) and more than100-fold higher in iNOS^−/−^ mice infected with 5 × 10^6^ *T. congolense* than in infected WT mice (b). WT mice infected with either dose survive for more than 30 days (the duration of observation period) (c). iNOS^−/−^ mice infected with 10^3^ *T*. *congolense* have a mean survival time of 14 ± 7 days and iNOS^−/−^ mice infected with 5 × 10^6^ *T. congolense* survive for only 6.8 ± 0.1 days (c).

BALB/c mice are highly susceptible, whereas C57BL/6 mice are relatively resistant to *T. congolense* infections [9, 28]. In iNOS^−/−^ C57BL/6 mice infected with 10^3^ *T. congolense*, the initial parasitemia was about 5-fold higher than in infected WT C57BL/6 mice (Figure 1(a)). In iNOS^−/−^ mice infected with 5 × 10^6^ *T. congolense*, the initial parasitemia was about 100-fold higher than in infected WT mice and, contrary to the infected WT mice, the iNOS^−/−^ mice infected with 5 × 10^6^ *T. congolense* did not control the parasitemia (Figure 1(b)). Whereas the WT mice infected with 10^3^ or 5 × 10^6^ *T. congolense* survived for more that 30 days (Figure 1(c)) (the duration of observation period). iNOS^−/−^ mice infected with 10^3^ *T. congolense *had a mean survival time of 14 ± 7 days and iNOS^−/−^ mice infected with 5 × 10^6^ *T. congolense *survived for only 6.8 ± 0.1 days (Figures 1(c) and 1(d)). These experiments suggest that the survival time of iNOS^−/−^ mice infected with *T. congolense* might be dose-dependent, a finding we have never observed in infections of wild-type mice in our previous work.

### 3.2. Decreased Production of Nitric Oxide and Enhanced Synthesis of Cytokines by Spleen Cell Cultures of Infected iNOS^−/−^ Mice

**Figure 2:**
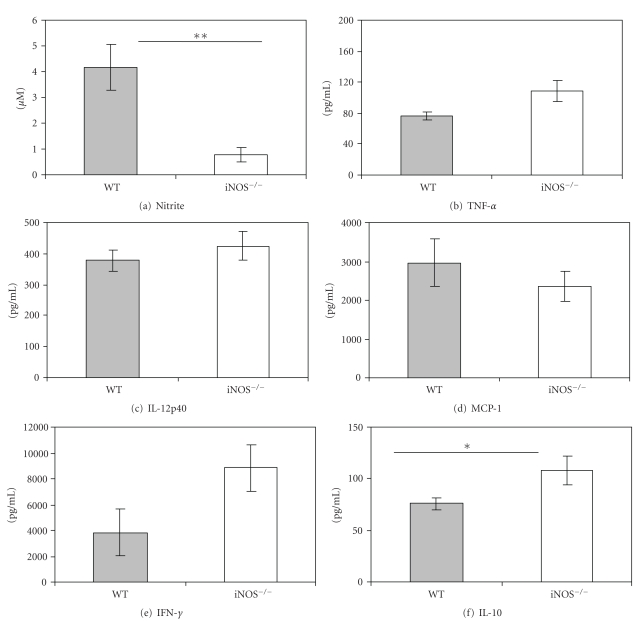
iNOS^−/−^ mice infected with 10^3^ *T. congolense* produce no detectable nitric oxide but more IL-10 than infected wild-type (WT) mice. Mice were infected with 10^3^ *T. congolense*. On day 6 postinfection, spleen cells were cultured at an optimal cell density (5 × 10^6^/mL) for 48 hours. Cytokines in culture supernatants were measured by ELISA, nitrite by the Griess reaction. *P < .05, **P < .01.

**Figure 3:**
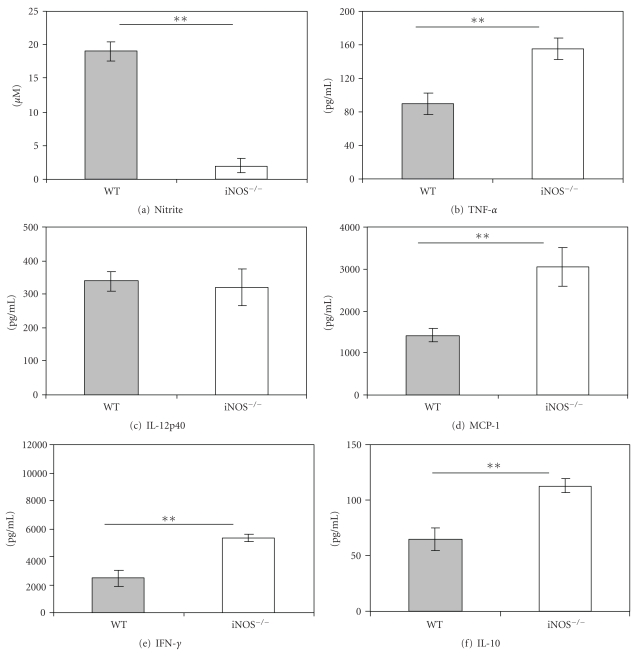
iNOS^−/−^ mice infected with 5 × 10^6^ *T. congolense* produce no detectable nitric oxide but more TNF-*α*, IL-10, and IFN-*γ* than infected wild-type (WT) mice. Mice were infected with 5 × 10^6^ *T. congolense*. On day 6 postinfection, spleen cells were cultured at an optimal cell density (5 × 10^6^/mL) for 48 hours. Cytokines in culture supernatants were measured by ELISA, nitrite by the Griess reaction. **P < .01.

Spleens collected on day 6 were cultured for the measurement of synthesis of NO and cytokines as determined by their levels in the supernatant fluids of the cultures. The measured levels of nitrite in spleen cell cultures from iNOS^−/−^ mice infected with either 10^3^ (Figure 2(a)) or 5 × 10^6^ (Figure 3(a)) parasites were below the sensitivity of the assay (2.5 *μ*M), whereas significant amounts of nitrite were measured in cultures from infected WT mice. There were no detectable differences between cytokine levels in cultures from iNOS^−/−^ versus WT mice infected with 10^3^ *T. congolense*, except for IL-10 which was significantly (*P* < .05) higher in cultures from iNOS^−/−^ mice (Figures 2(b)–2(f)). The levels of TNF-*α* (Figure 3(b)), MCP-1 (Figure 3(d)), IFN-*γ* (Figure 3(e)), and IL-10 (Figure 3(d)) were significantly (*P* < .01) higher in cultures from iNOS^−/−^ than in those from WT mice infected with 5 × 10^6^ *T. congolense*. There were no detectable differences in the levels of IL-12p40 (Figure 3(f)). Thus, early death of infected iNOS^−/−^ mice is associated with decreased production of NO but enhanced levels of cytokine synthesis correlated with the degree of parasitemia (Figures 1(a) and 1(b)).

### 3.3. L-NIL Given Intravenously at Doses of 20 or 40 mg/kg Body Weight Did Not Enhance Parasitemia of Infected Mice

**Figure 4:**
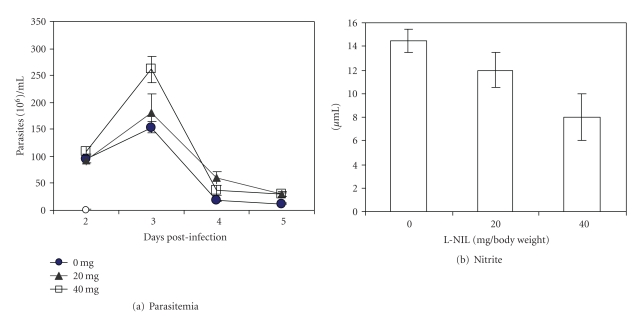
Treatment of mice with L-NIL, an iNOS-specific inhibitor, had no effect on parasitemia but a moderate effect on the production of NO in spleen cell cultures. Groups of 6 C57BL/6 mice were treated i.v. with L-NIL (20 or 40 mg/ body weight) on day −1, 1, 2, and 3 postinfection. Mice were infected i.p. with 5 × 10^6^ *T. congolense* TC13 on day 0. Parasitemia (a) was determined. On day 6 postinfection, spleen cells were cultured at an optimal cell density (5 × 10^6^/mL) for 48 hours. Nitrite (b) in culture supernatants was measured by the Griess reaction. **P ≤ .01; ***P ≤ .001.

L-N6-(1-iminoethyl) lysine (L-NIL) has been shown to be a specific inhibitor of iNOS [25]. This iNOS inhibitor has been given in the drinking water [29] or by injection [30, 31]. We chose to administer L-NIL at dosages of 20 or 40 mg/kg body weight intravenously on days −1, 1, 2, and 3. We expected that this treatment would enhance parasitemia since this regimen of administration of L-NIL was effective in a different experimental design of *T. congolense* infection [22] (see Discussion). At these dose regimens, however, we did not observe an effect on the parasitemia (Figure 4(a)) nor complete inhibition of the synthesis of induced NO by spleen cell cultures of the L-NIL-treated, infected mice (Figure 4(b)). Thus, it appeared that synthesis of iNOS was not effectively inhibited by L-NIL in mice in the presence of high levels of *T. congolense*.

### 3.4. Nitric Oxide Inhibits Multiplication of *T. congolense* In Vivo

**Figure 5:**
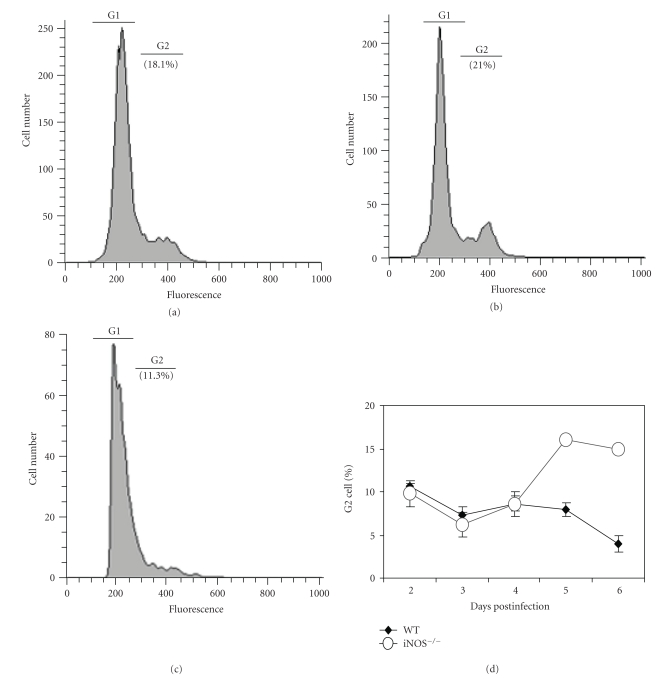
Higher rate of multiplication of *T. congolense* in infected iNOS^−/−^ mice than in infected wild-type mice. Groups of 6 WT C57BL/6 and iNOS^−/−^ mice were infected with 5 × 10^6^ *T. congolense* TC13. The percentage of trypanosomes being in theG1 and S/G2/M phases was determined by DNA FACS analysis (see Materials and Methods). (a) A typical FACS DNA pattern of *T. congolense* freshly isolated from infected immunosuppressed mice. (b) A typical FACS DNA pattern of *T. congolense* in infected iNOS^−/−^ mice at day 6 postinfection. (c) A typical FACS DNA pattern of *T. congolense* in infected WT C57BL/6 mice. (d) Kinetics of S/G2/M phases of trypanosomes in WT and iNOS^−/−^ mice following TC13 infection. Note: on days 5 and 6, the trypanosomes in iNOS^−/−^ mice had a significantly higher ratio of organisms in the S/G2/M phases than trypanosomes in WT mice.

By using simple, reliable methods for staining nuclear DNA, cell cycle compartment analysis can be used to measure cell proliferation. We decided to measure potential differences of proliferation of trypanosomes in infected iNOS^−/−^ and WT mice by using DNA staining with propidium iodide, and subsequent FACS analysis [26, 27]. We initially purified trypanosomes from immunosuppressed CD1 mice, stained them with propidium iodide and analyzed them by FACS to establish the gating for determining the ratios of S+G2+M to S1 phases of the trypanosomes (Figure 5(a)). We then determined the ratios of S+G2+M to S1 phases of the trypanosomes in the blood of iNOS^−/−^ and WT mice infected with 5 × 10^6^ parasites. We found significantly higher ratios of S+G2+M to S1 phases in the blood stream trypanosomes of iNOS^−/−^mice on days 5 and 6 (Figure 5(d)). These findings indicate that the trypanosomes in the iNOS^−/−^ mice had a higher rate of proliferation than the ones in WT mice. These results lead us to the conclusion that one of the mechanisms of controlling the parasitemia is due to the cytotoxic effect of NO.

### 3.5. Infected CD11b^−/−^ Mice Have Enhanced Nitric Oxide Production and Enhanced Control of First Parasitemia

**Figure 6:**
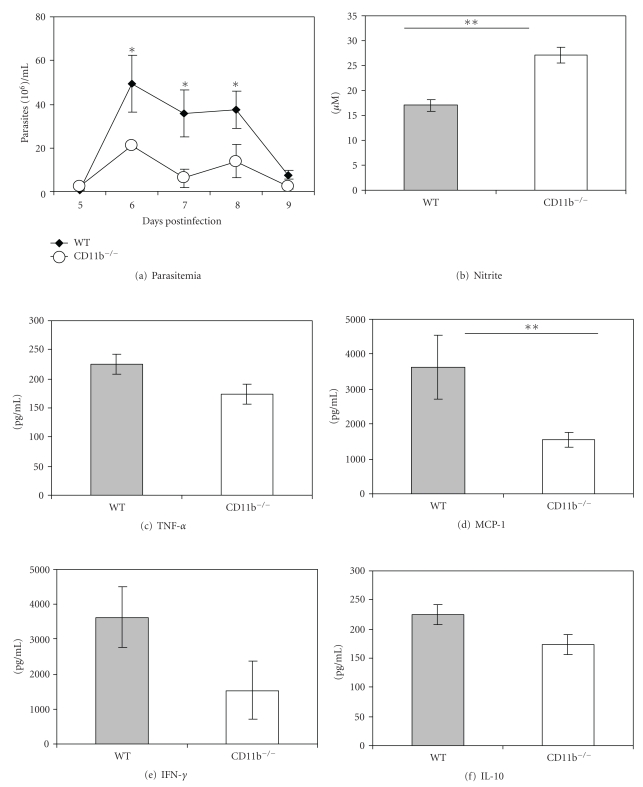
CD11b^−/−^ mice infected with* T. congolense *have a higher production of nitric oxide and show better control of the initial parasitemia than infected wild-type mice. Mice were infected with 10^3^ *T. congolense* TC13. The first parasitemia was shown to be significantly lower in infected CD11b^−/−^ mice than in infected wild-type (WT) mice (a). Spleen cell cultures (day 6) of infected CD11b^−/−^ mice produced significantly more nitrite than spleen cell cultures of infected WT mice (b). Cytokines in the supernatants of spleen cell cultures of CD11b^−/−^ mice and wild-type mice 6 days after infection with* T. congolense *(c–f). Note: Infected CD11b^−/−^ mice had a significant decrease in production of MCP-1 (d) but no detectable differences in production of TNF-*α* (c), IL-10 (f), or IFN-*γ* (e). *P < .05, **P < .01.

We have previously shown that IgM anti-VSG-mediated phagocytosis by macrophages induced more NO in macrophages obtained from CD11b^−/−^ mice than in those from wild-type mice. We thus tested the parasitemia in CD11b^−/−^ and wild-type mice infected with 10^3^ *T. congolense*. The first parasitemia was significantly lower in the CD11b^−/−^ mice and the spleen cell cultures of the CD11b^−/−^ mice produced more NO but less MCP-1 than those of the infected wild-type mice (Figure 6). The subsequent parasitemias were not significantly different in the infected two mouse strains (not shown). Considering that most phagocytosis of trypanosomes during the first parasitemia is due to IgM anti-VSG antibody, the above observations would suggest that the effect of NO on *T. congolense* is more powerful in controlling parasitemia of *T. congolense* than phagocytosis mediated by IgM anti-VSG.

## 4. Discussion

In iNOS^−/−^ mice infected with *T. congolense*, parasitemia was significantly enhanced and survival time dramatically reduced (Figure 1), providing strong support for a significantly protective role of inducible NO in infections by *T. congolense*. Thus, these results confirm the findings reported by Magez et al. [21]. The degree of disease in *T. congolense*-infected iNOS^−/−^ mice appears to be correlated with the dose of infection (Figures 1, 2, and 3), a condition we have never observed in infected WT mice. In WT mice, we merely observe an inverse correlation of the dose of infection and the prepatent period (Figures 1(a) and 1(b)) [32]. In iNOS^−/−^ mice infected with a high dose of *T. congolense*, production of nitric oxide was very low but synthesis of cytokines (TNF-*α*, MCP-1, IL-10, and IFN-*γ*) by spleen cell cultures was significantly enhanced (Figure 3). We interpret the increased levels of cytokines to be an expression of enhancement of the systemic inflammatory response syndrome described by us previously [33, 34].

Since induced NO exhibits such a crucial role in the control of parasitemia and survival, it is plausible to assume that infected WT C57BL/6 mice should become susceptible to disease if treated with the specific iNOS inhibitor L-NIL. Surprisingly, inhibition of iNOS by L-NIL given at doses of 20 or 40 mg/kg body weight i.v. to infected WT C57BL/6 mice at days −1, 1, 2, and 3 neither effectively inhibited all NO synthesis in the spleen nor enhanced parasitemia of infected mice. A similar treatment was effective in a different experimental design. We found that previous treatment with an optimal amount of anti-CD25 antibody of susceptible BALB/c mice subcutaneously infected with *T. congolense* led to prevention of parasitemia and disease in the infected mice [22]. This condition could be reversed by treatment with L-NIL [22]. We presently have no sufficient explanation for the discrepancy of results obtained with iNOS^−/−^ mice and iNOS inhibitor in this study. We speculate that the presence of high levels of *T. congolense* might, by a yet unknown mechanism directly or indirectly, interfere with the iNOS-inhibiting effect of L-NIL.

NO has been shown to prevent African trypanosomes to grow in vitro [14, 17]. Thus judging from the experiments with iNOS^−/−^ mice, it is plausible to assume that NO does inhibit the multiplication of *T. congolense* in vivo. Since the G2 : G1 ratios of cells provide insight into the rate of multiplication of cells [26, 27], we proceeded to determine the percentages of the blood stages of *T. congolense *that were in the S/G2/M versus G1 phase. We did observe a significantly higher ratio of the S/G2/M phases in *T. congolense* obtained from the blood of infected iNOS^−/−^ mice (Figure 5), indicating that the parasites had a higher multiplication rate in the iNOS^−/−^ mice than in the WT mice. From these observations, we conclude that induced nitric oxide inhibits multiplication of *T. congolense* in infected WT mice in vivo. The experiments do not tell us where the interaction of trypanosomes and NO might occur. Thus we might ask the question: where does secreted NO interact with the intravascular trypanosomes? Phagocytosis of trypanosomes sensitized by anti-VSG antibodies takes place by macrophages in the liver and spleen [5, 6, 8]. Macrophages are also a major source of induced NO upon phagocytosis of trypanosomes [7, 14, 17]. The cytotoxic effect of NO secreted by macrophages in vivo will be effective at a very short range only, since NO is efficiently neutralized by hemoglobin of red blood cells [35]. During early infection, there is a stage of the host response when plasma levels of IgG anti-VSG antibodies are still too low to mediate phagocytosis of trypanosomes. It is conceivable that, at this stage, IgG anti-VSG antibodies, especially IgG2a anti-VSG antibodies [14, 36], might nevertheless be present in sufficient concentration to mediate temporary adherence of trypanosomes to macrophages. We propose that this process might result in bringing trypanosomes into close apposition to macrophages, that is, to Kupffer cells in the liver [5, 6, 8] and to macrophages in the marginal zone of the spleen [37]. Thus, released NO, in turn, could be sufficiently cytotoxic to the temporarily caught parasites to inhibit their multiplication. This might be analogous to the control of growth of *T. lewisi* and *T. musculi* [38, 39].

We previously found that IgM anti-VSG antibody-mediated phagocytosis of *T. congolense* led to higher NO synthesis by CD11b^−/−^ macrophages than by WT macrophages [7]. From those experiments, we concluded that signaling of inhibition of NO synthesis in WT macrophages might occur via complement receptor CR3 and not by ingested antigen intracellularly. We further concluded that this process is complement-independent. Molecules of other microbes have been shown to bind in complement-independent fashion to the lectin site of CR3, that is different from the binding site of iC3b [40–42]. We found that IgM anti-VSG induces shedding of soluble VSG (sVSG) from *T. congolense* (Pan et al., unpublished). We speculated that the glycan of the sVSG [2] might bind to the lectin site of CR3. It is relevant to note that Coller et al. [43] found that IFN-*γ*-induced NO production by a macrophage cell line was reduced after the macrophages were exposed to sVSG of *T. brucei rhodesiense.* In this context, we infected CD11b^−/−^ mice with *T. congolense*. We found enhanced nitric oxide production and enhanced control of the first parasitemia, but not of the later parasitemias, in the infected CD11b^−/−^ mice as compared to the infected WT mice. The first parasitemia is predominantly controlled by IgM anti-VSG antibodies. Thus, it would appear that the cytotoxic effect of NO on *T. congolense* is more powerful in controlling parasitemia of *T. congolense* than phagocytosis mediated by IgM anti-VSG. It also appears that African trypanosomes have developed a mechanism to evade the deleterious effect of NO by inducing an IgM anti-VSG-mediated inhibition of NO synthesis [7, 14]. 

 In summary, we conclude that in mice infected with *T. congolense*, induced NO contributes to control of parasitemia by inhibiting the growth of the trypanosomes.

## Abbreviations

iNOS: Inducible nitric oxide synthase
NO: Nitric oxide
VSG: Variant surface glycoprotein
L-NIL: L-N6-(1-imminoethyl) lysine.
